# Prevalence and risk factors of chronic kidney disease in an HIV positive Mexican cohort

**DOI:** 10.1186/s12882-021-02526-4

**Published:** 2021-09-23

**Authors:** Verónica Valdivia-Cerda, Monserrat Alvarez-Zavala, Karina Sánchez-Reyes, Rodolfo I. Cabrera-Silva, Vida V. Ruiz-Herrera, Aldo D. Loza-Salazar, Pedro Martínez-Ayala, Juan C. Vázquez-Limón, Guillermo García-García, Jaime F. Andrade-Villanueva, Luz A. González-Hernández

**Affiliations:** 1 Servicio de Nefrología, Hospital Civil de Guadalajara “Fray Antonio Alcalde”, Guadalajara, Jalisco Mexico; 2 Universidad de Guadalajara, Instituto de Investigación en Inmunodeficiencias y VIH (InIVIH), Centro Universitario de Ciencias de La Salud, Jalisco, México; 3 Hospital Civil de Guadalajara “Fray Antonio Alcalde”, Unidad de VIH, Guadalajara, Jalisco Mexico

**Keywords:** Chronic kidney disease, HIV-positive, Mexican cohort, Antiretroviral therapy

## Abstract

**Background:**

HIV subjects have several kidney pathologies, like HIV-associated nephropathy or antiretroviral therapy injury, among others. The global prevalence of Chronic Kidney Disease (CKD) is 8–16%; however, in HIV subjects, the prevalence varies between geographic regions (2–38%). The aim was to determine the prevalence of CKD and identify the associated risk factors.

**Methods:**

A longitudinal descriptive study was carried out at the 'Hospital Civil de Guadalajara' Feb'18 – Jan'19. Basal clinical, demographic, opportunistic infections (OI), and laboratory data were obtained at months 0 and 3; inclusion criteria were ≥ 18 years old, naïve HIV + , urine albumin/creatinine ratio, serum creatinine & urine test, and signed informed consent. Descriptive and multiple logistic regression statistical analyses were made.

**Results:**

One hundred twenty subjects were included; 92.5% were male, 33 ± 9.5 years, 60% consumed tobacco, 73% alcohol, and 59% some type of drug. The CKD prevalence was 15.8%. CKD patients had a higher risk of hepatitis C virus coinfection, Relative Risk (RR):5.9; HCV infection, RR:4.3; ≥ 30 years old, RR:3.9; C clinical-stage, RR:3.5; CD4^+^ T cells count < 200 cells/μL, RR: 2.4; and HIV-1 viral load ≥ 100,000 cop/mL, RR: 2.7.

**Conclusions:**

Our study showed a higher CKD prevalence in patients with HIV; higher CKD development with coinfections as Hepatitis C Virus and *Mycobacterium tuberculosis*. The identification and prompt management of CKD and coinfections should be considered to avoid the progression and to delay renal replacement therapy as long as possible.

## Background

The life expectancy of people with HIV infection has been improving with antiretroviral therapy (ART) usage. However, chronic kidney disease (CKD) denotes one of the leading health concerns among HIV-infected subjects, as it is a common complication for these patients, secondary to HIV itself or by the kind of ART used or derivable of the growing burden of traditional risk factors associated with CKD [1].

CKD is defined by an estimated glomerular filtration rate (eGFR) < 60 mL/min/1.73 m^2^ or one or more of the following markers of kidney damage (persistent through 3 months): a) albuminuria (Albumin excretion rate ≥ 30 mg/day, albumin-creatinine ratio (ACR) ≥ 30 mg/g), b) urinary sediment abnormalities, c) electrolyte and other abnormalities due to tubular disorders, d) abnormalities detected by histology, e) structural abnormalities detected by imaging or f) history of kidney transplantation [2].

The global prevalence of CKD is 8–16%, while, in HIV subjects, the prevalence varies between geographic regions (in a range from 2 to 38%) [1]. A study in Northwest Mexico reported a prevalence of 11.7%, higher than the one reported in North America and Europe (4.7 and 9.7%, respectively) [3]. Thereby, the prevalence of CKD varies clearly across populations [4].

HIV subjects on ART achieve viral replication suppression with an improvement of immunological reconstitution. However, they may suffer from premature aging and a continuous chronic inflammation, which induces metabolic disorders and non-communicable diseases (e.g., hyperlipidemia, diabetes, hypertension, and abnormal body fat composition), conditions also related to CKD [5].

HIV subjects have several kidney pathologies, such as a) Noncollapsing focal-segmental glomerulosclerosis, b) HIV-associated nephropathies (classic HIVAN or Lupus-like glomerulonephritis), c) Immune-complex kidney disease, d) IgA nephropathy, e) Thrombotic microangiopathy, f) Membranoproliferative glomerulonephritis, g) Membranous glomerulonephritis, h) Kidney injury by ART consumption (tubulointerstitial nephritis, crystal nephropathy, and tenofovir disoproxil fumarate–induced nephrotoxicity (Fanconi's syndrome)), i) By Opportunistic Infections (OI), like Mycobacterial infections, Cytomegalovirus, *Mycoplasma, Microsporidia*, Bacterial pyelonephritis, among others; j) By coinfections, such as hepatitis C virus (HCV), hepatitis B virus (HBV), Herpes simplex, or k) By infiltrative kidney lesions secondary to lymphoma or Kaposi's sarcoma, among others [6, 7].

In patients with HIV infection, the equations for calculating the eGFR have been evaluated, showing good accuracy. The Chronic Kidney Disease Epidemiology Collaboration (CKD-EPI) equation, which incorporates serum creatinine level and demographic aspects, appears to provide the most accurate estimates among HIV-infected persons who are stable on ART. Nonetheless, it is necessary to be aware that some drugs frequently used in these patients, such as trimethoprim, cobicistat, and HIV integrase inhibitors like dolutegravir, can inhibit proximal renal tubular secretion of creatinine, which may increase serum creatinine concentration altering the eGFR results [7].

This study aimed to determine the prevalence of CKD in a naïve HIV-positive population from western Mexico, using the CKD-EPI equation ± markers of kidney damage to identify the risk factors associated with CKD.

## Methods

A longitudinal descriptive study was carried out at the HIV unit of the 'Hospital Civil de Guadalajara' in western Mexico. The patients were evaluated from February 1st., 2018 to January 31st., 2019. Subjects were invited to voluntarily participate in the study the same day of their first clinic visit at the HIV unit, in which the fulfillment of the selection criteria was determined. The inclusion criteria were: naïve HIV positive (HIV +), men and women ≥ 18 years, with available urine albumin-creatinine ratio, serum creatinine, and urinalysis at baseline were included. All patients signed an informed consent. Basal sociodemographic, clinical, OI, and laboratory data were obtained and reassessed at months 3, 6, and 12. The study was approved by the Hospital's Ethics Committee (Register number: 211/18).

Sociodemographic and clinical variables included gender, age, ethnic group, academic level, marital status, comorbidities, and their medications, ART indicated at their first medical visit, smoking, alcohol, and drug abuse. Participants were asked whether they had a physician's diagnosis of any of the 19 chronic diseases described in the World Health Organization's International Classification of Diseases (ICD-10) [6]. Clinical stage of HIV infection (subdividing them into those without and those with clinical features of AIDS) according to the CDC classification [7] to identify OI. The HIV 1-RNA viral load in plasma was measured with the ROCHE Amplicor HIV-1 Monitor 1.5 Ultrasensitive PCR technique with COBAS AmpliPrep/ Cobas Taqman. Controlled HIV infection was considered if the viral load was ≤ 40 copies/mL for at least six months on treatment. The absolute CD4^+^ T cells count (cells/μL), nadir CD4^+^ T cells count and percentage, absolute CD8^+^ T cells count (cells/μL), and the CD4/CD8 ratio were evaluated with FACSCalibur System Beckton Dickinson BD.

Additionally, urine albumin/creatinine ratio (ACR) was measured with the Clinitek Microalbumin 2 reagent strips, Siemens Healthineers. eGFR was estimated with the CKD-EPI equation [8]. Serum creatinine (sCr), urinalysis, serum lipid levels, serum bilirubin, liver enzymes, complete blood count, serum glucose, serum electrolytes, and Body Mass Index (BMI) were analyzed and compared between patients with and without CKD.

CKD was defined as an eGFR < 60 mL/min/1.13 m^2^, and/or ACR ≥ 30 mg/g or hematuria, present for > 3 months. CKD staging was determined by the Kidney Disease Improving Global Outcomes (KDIGO) recommendations [9].

Median and interquartile ranges (IQR) or mean and standard deviations (SD) were reported depending on data distribution, using the Shapiro-Wilks normality test to determine Gaussian or non-Gaussian distribution. According to the variable analyzed, the data were compared using either Student's t-test, Fisher exact, or Chi-square. Spearman's rho correlation coefficient was used. Relative Risks (RR) were reported and, a multiple logistic regression statistical analysis was performed to estimate the association of CKD presence, considering the following variables: ART usage (tenofovir + or –, protease inhibitors + or –), age (> or ≤ 30 years old), gender, school grade, clinical stage, CD4^+^ T cells count (> or ≤ 200 cells/μL), viral load (> or ≤ 100,000 copies/mL), low CD4/CD8 ratio (< 0.3), HCV, VHB, or syphilis coinfections, tuberculosis (TB) infection, serum albumin levels, BMI, concomitant drugs, and tobacco, alcohol, or drugs use. Cox and Snell R-squared are reported. For all the analyses, a *p*-value of < 0.05 was considered significant. Data were analyzed using Statistical Package for the Social Scientist (SPSS) version 23 (IBM) and GraphPad Prism 7 software.

## Results

From February 1st. 2018 to January 31st. 2019, a total of 120 subjects were included. Their mean age was 33 ± 9.5 years; 111 (92.5%) patients were male, with a mean BMI of 23.1 ± 4.7 kg/m^2^. Sixty percent smoked, 73% consumed alcohol, and 59% were drug abusers. Moreover, 4.2% had HCV coinfection, 2.7% had HBV coinfection, and 7.5% had TB infection. Regarding comorbidities, 2.5% had type 2 diabetes mellitus, and 0.8% had systemic arterial hypertension. Only 92 subjects completed their follow-up appointments, 15.8% were lost to follow-up, and 9 (7.5%) participants died during the study period.

The 30.4% (28/92) of patients were in AIDS stage according to HIV CDC Classification, with a median of CD4^+^ T cell count of 247 cells/μL (IQR: 109—386 cells/μL); furthermore, 72% had CD4/CD8 ratio ≤ 0.20 and only 7.8% had an optimum CD4/CD8 ratio (≥ 8). The median viral load was 70,750 copies/mL (IQR:26,470 – 221,000 copies/mL).

ART was started as a Single-Tablet Regimen (STR) in 87.5% of the participants; 51.7% based on Nonnucleoside Reverse Transcriptase Inhibitors (NNRTIs): Tenofovir Disoproxil Fumarate (TDF)/Emtricitabine (FTC)/Efavirenz (EFV). The remaining ARTs (35.8%) were based on Integrase Inhibitors (INsTI), Abacavir (ABC)/Lamivudine (3TC)/Dolutegravir (DTG) or TDF/FTC/Elvitegravir/ Cobicistat (EVG/COBI). The median in days for starting ART was 20 days (IQR: 12 – 26), and 95% achieved undetectability after three months of treatment.

**Fig. 1:**
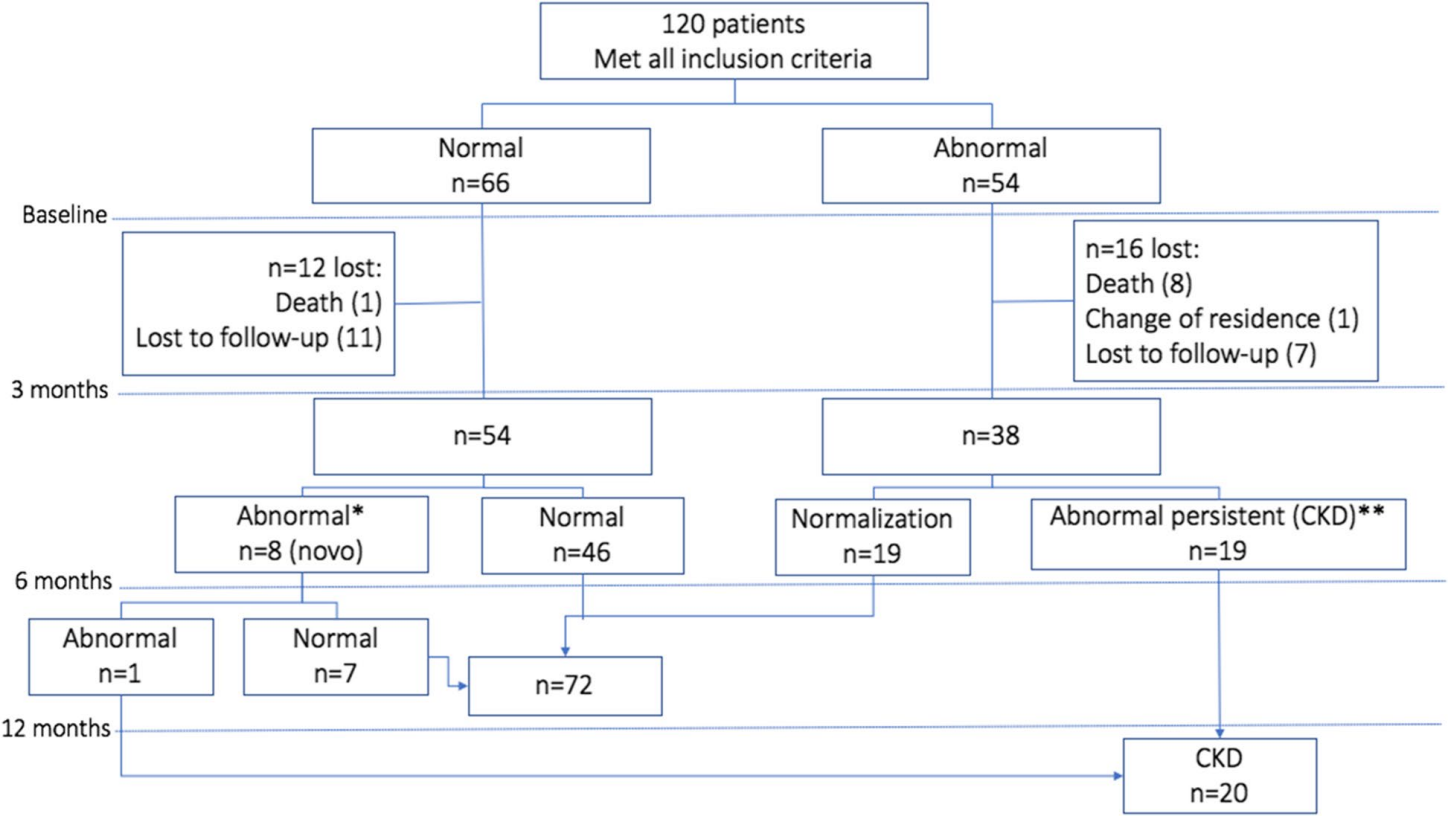
Diagram of participants recruitment and follow up over a year. *One patient with persistent hematuria by nephrolithiasis. ** Persistent abnormal ACR; 1 patient with Membranous Glomerulopathy, 1 patient with Focal Segmental Glomerular Sclerosis Glomerulonephritis, and 1 patient with Hematuria

At baseline, 54 (45%) patients had abnormal kidney function tests. After one year of follow-up, 19 patients remained with CKD (Fig. 1). One more patient developed CKD during the surveillance period, but the patient was already on ART; hence, it was not included in our analysis. The prevalence of CKD in naïve patients in our population was 15.8% (19/120). One subject had persistent hematuria, another had abnormal urine ACR (> 3 mg/mmoL) plus low eGFR (20 mL/min/1.73 m^2^), and 17 patients had abnormal urine ACR (> 3 mg/mmoL). Two patients underwent kidney biopsy, one because of a rapid deterioration of kidney function with a diagnosis of focal-segmental glomerulosclerosis. The other was due to nephrotic secondary to membranous glomerulonephritis plus HCV coinfection. The CKD stage more frequently seen in our population was G1A2 stage (95%).

Patients with CKD were older (34 (IQR: 31–44) vs. 30 (IQR: 26–36) years, *p* = 0.028); had higher HIV-1 viral load (169,000 (IQR: 73,700–453,847) cop/mL vs. 52,000 (IQR: 24,650–111,000) cop/mL, *p* = 0.0001), lower CD4^+^ T cells count (163 (IQR: 58–300) cells/μL vs. 323 (IQR: 161–552) cells/μL; *p* = 0.003), lower CD4/CD8 ratio (0.20 (IQR: 0.09–0.33) vs. 0.30 (IQR: 0.14–0.51) *p* = 0.022), and more advanced CKD stage (47% in stage C and 79% with CD4^+^  < 200 cells/μL) than non-CKD patients (Table 1).

**Table 1:** Clinical, laboratory, and HIV-infection characteristics in patients with CKD and without it

Variable	Without CKD n = 73	With CKD n = 19	Relative Risk (95%CI)	*P-*value
Age (years) median/IQR	30 (26–36)	34 (31–44)	-	0.028
Age > 30 years (n/%)	37 (50)	16 (84)	3.9 (1.2–13)	0.008
Gender				
Female (n/%)	4 (6)	1 (5.5)	0.87 (0.14–5.3)	ns
Male (n/%)	69 (94)	18 (94.5)	0.88 (0.14–5.4)	ns
Tobacco (n/%)	42 (57)	13 (68)	1.5 (0.61–3.5)	ns
Drugs (n/%)	41 (56)	11 (59)	1.1 (0.47–2.4)	ns
Alcohol (n/%)	56 (77)	15 (78)	1.1 (0.41–3)	ns
Type 2 Diabetes Mellitus (n/%)	3 (4.1)	0	-	-
Arterial hypertension	0	1 (5.2)	-	-
HIV-1 Viral Load (cop/mL) Median/IQR	52,000 (24,650–111,000)	169,000 (73,700–453,847)	-	0.001
Viral Load HIV-1 (≥ 100,000 cop/mL)	24 (32)	12 (63)	2.7 (1.2–6.1)	0.019
CD4^+^ T cells count (cells/μL) Median/IQR	323 (161–552)	163 (58–300)	-	0.003
CD4^+^ T cells < 200 cells/μL (n/%)	32 (43)	15 (79)	4 (1.2–13)	0.007
CD4/CD8 ratio (median/IQR)	0.30 (0.14–0.51)	0.20 (0.09–0.33)	-	0.022
Clinical stage at baseline:				
Stage A	43 (58.9)	7 (36)	0.56 (0.24–1.3)	ns
Stage B	20 (27.4)	3 (15.7)	0.56 (0.18–1.8)	ns
Stage C	10 (13.7)	9 (47.3)	3.5 (1.9–7.3)	0.002
Coinfections				
Hepatitis B virus	2 (2.7)	0	-	-
Hepatitis C virus	2 (2.7)	4 (21)	3.8 (1.8–7.9)	0.015
*M. tuberculosis*	2 (2.7)	5 (26)	4.3 (2.2–8.5)	0.003
Syphilis (VDRL +)	23 (32)	5 (26)	0.82 (0.33–2)	ns

*IQR* Interquartile range, *CI* Confidence interval

Risk factors for CKD were the presence of TB infection (RR: 4.3 (95%CI: 2.2–8.5; *p* = 0.003), and HCV coinfection (RR: 3.3 (95%CI: 1.4–7.6; *p* = 0.05). By multivariate logistic regression analysis, age > 30 years, high viral load (> 100,000 copies/mL), low CD4^+^ T cells count (< 200 cells/μL), HCV coinfection, and TB infection were associated to the development of CKD (Cox and Snell R-squared: 0.471; *p* = 0.001). Interestingly, a low CD4/CD8 ratio, concomitant drugs, and the type of ART used were not associated with CKD development (Table 2).

**Table 2:** Univariate and multivariate analysis of risk factors associated with CKD

Variable	Univariate Relative Risk (95%CI)	*P-*value	Multivariate Relative Risk (95%CI)	*P-*value
Age > 30 years (n/%)	3.9 (1.2–13)	0.008	8.47 (1.5–46.8)	0.014
HIV-1 Viral Load (≥ 100,000 copies/mL)	2.7 (1.2–6.1)	0.019	4.6 (1.2–16.9)	0.020
CD4^+^ T cells < 200 cells/μL (n/%)	4 (1.2–13)	0.007	5.2 (1.2–12)	0.011
Clinical stage C	3.5 (1.9–7.3)	0.002	-	ns
Coinfections:				
Hepatitis C virus (HCV)	3.8 (1.8–7.9)	0.015	5.6 (3.6–9.4)	0.001
*M. tuberculosis*	4.3 (2.2–8.5)	0.003	33 (1.92–56.5)	0.016

Multivariate analysis considering the following variables: use of Antiretroviral Therapy (ART), age, gender, school grade, clinical stage, level of CD4^+^ T cells, level of viral load, CD4/CD8 ratio, *HCV* Hepatitis B Virus (HBV) or Syphilis coinfections, Tuberculosis infection, serum albumin levels, Body Mass Index (BMI), concomitant drugs, tobacco, alcohol, or drugs use. Cox and Snell R-squared: 0.471; *p* = 0.001. *CI* Confidence interval

Finally, we found a positive correlation between the age and the ACR (r = 0.326; *p* = 0.003), as well as a negative correlation between age and eGFR (r = -0.461; *p* = 0.0001) and between age and serum albumin level (r = -0.419; *p* = 0.0001).

## Discussion

A higher prevalence (15.8%) was identified in our study compared to global CKD prevalence (8%) and previous reports in the HIV Mexican population (11.7%) [1, 3]. Among the well-recognized risk factors for the development of CKD reported in previous studies are HIV infection itself, the use of nephrotoxic drugs (like ARTs, mainly with TDF, abacavir/lamivudine, and atazanavir), a low CD4^+^ T cells count, the presence of comorbidities, among others [3–5]. In our study, the main risk factors associated with CKD development were age > 30 years old, more advanced HIV disease (with high viral load and lower CD4^+^ T cells count), and TB and HCV coinfections. An interesting finding in our study is the relatively mild CKD stage detected in our patients. Similar to our report, a study performed in Burundi found that 2% of the patients had an eGFR < 60 mL/min/1.73 m^2^, plus urinary abnormalities were more common [10]. In a cross-sectional report in Mexican patients, 37.5% were diagnosed with CKD using albuminuria alone [3]. Possible explanations for the larger proportion of early stages of CKD in our study are its prospective design, the relatively short follow-up time, and that they were all ATR naïve at the onset, which is a risk factor for CKD development in the long term.

Additionally, 5 (20%) patients with CKD received *Pneumocystis jirovecii* pneumonia prophylaxis with trimethoprim-sulfamethoxazole. This drug may cause an elevation of serum creatinine by inhibiting tubular creatinine secretion. It can affect the estimation of GFR, leading to the misperception of a more severely affected kidney function than it actually is. Furthermore, this drug could potentially increase the ACR as it only affects creatinine secretion but not albuminuria [11].

Regarding this study, it should be noted that four out of five patients taking trimethoprim-sulfamethoxazole improved their ACR levels in the third month compared to their baseline levels, while the fifth maintained the same ACR from baseline levels. None of the patients had an altered GFR.

HIV itself can produce nephropathy through a direct podocyte and tubular cell infection; podocyte infection causes injury and dedifferentiation, with loss of kidney function, whereas tubular cell infection is associated with mitochondrial dysfunction and tubulointerstitial inflammation [12]. Also, it has been described that Vpr (an HIV protein) induces apoptosis of tubular epithelial cells via the activation of caspase 8 through stimulation sustained by extracellular signal-regulated MAP kinases [13]. Consequently, a high viral load could provoke more kidney damage, as identified in this study, where viral load > 100,000 copies/mL showed to be a risk factor for CKD development in both the univariate and multivariate analysis (RR: 2.7; *p* = 0.019 and RR: 4.6; *p* = 0.020, respectively).

Low CD4^+^ T cell count has been associated with renal disease in HIV-positives patients. A study found 3.5-fold increased risk of albuminuria in subjects with CD4^+^ T cell count < 350 cells/μL [14]. In our study, 51% of the patients had CD4^+^ T cell count < 200 cells/μL, which was significantly more frequent in CKD patients. On the other hand, a low CD4/CD8 ratio has been used as a biomarker for disease progression of several non-AIDS-associated pathologies, such as kidney disease, heart disease, neurocognitive disorders, among others. Ratios between 1.5 and 2.5 are generally considered normal values. Low CD4/CD8 ratios are associated with altered immune function, chronic inflammation, and immunosenescence [15]. In our study, patients with CKD had a lower CD4/CD8 ratio than non-CKD patients (*p* = 0.022). Furthermore, it is worrying that, from the total of participants, 39% had a CD4/CD8 ratio < 0.30, considered as a marker of profound immune damage, with a high risk of non-AIDS-defining events or death [16]; plus, only 7.8% of all patients had an optimal CD4/CD8 ratio of ≥ 0.8 (data not shown).

TB infection is associated with several risk factors, including extremes of age, sex, malnutrition, immune alterations, comorbidities like HIV infection, diabetes mellitus, malignancy, organ transplantation, and CKD. This last condition itself increases 4.5 times the risk of TB compared to the general population. On the other hand, there is evidence that TB can also induce CKD due to tubulointerstitial nephritis through the formation of chronic granulomas. Also, around 8–15% of patients with pulmonary TB develop a genitourinary infection due to the hematogenous spread of the infection. Furthermore, some therapeutic agents used for TB infection are nephrotoxic, like rifampicin, isoniazid, and ethambutol.

It is known that a tuberculous kidney can develop a gross anatomic distortion secondary to calcifications, which decreases the glomerular filtration and, finally, provokes CKD. However, there is little evidence about the earlier renal changes in adult patients with TB and HIV coinfection, like the albuminuria demonstrated in our study [17].

Due to all of this, it has been described 1.27-folds more CKD incidence in patients with TB [18]. In our study, TB infection showed to be a high-risk factor for the development of CKD (RR:4.3; *p* = 0.003 (univariate analysis), RR:33; *p* = 0.016 (multivariate analysis)).

HCV infection is recognized as a risk factor in developing several extrahepatic affectations, including kidney disease. The main type of HCV-related glomerulonephritis is Type 1 membranoproliferative glomerulonephritis (MPGN), associated with type 2 cryoglobulinemia, while non-cryoglobulinemic MPGN, membranous glomerulonephritis, and focal segmental glomerular sclerosis are less frequent [19]. The principal mechanism for the development of these glomerulopathies is, through the deposition of immune complexes in the glomeruli (in the mesangium and capillary walls), with prominent matrix and mesangial cells, hypercellularity (mononuclear and polymorphonuclear leukocytes), and intracapillary thrombi; sometimes, small and medium-sized renal arteries vasculitis can be present. The prevalence of CKD is significantly higher in HCV patients (9.6 vs. 5.1%) and, mainly in these patients, the development of CKD and end-stage renal disease occur in a shorter time; furthermore, a higher viral load has been described as an independent predictor of CKD [17]. Nowadays, there are many drugs to treat HCV satisfactorily; thus, it is a priority to make an early diagnosis and offer the proper treatment, removing this risk factor, in addition to the benefit of stopping the liver damage [20]. In this study, HCV coinfection showed to be a significant risk factor for CKD development (RR:3.8; *p* = 0.015 (univariate analysis), RR:5.6; *p* = 0.001 (multivariate analysis)).

In 2014, the Joint United Nations Programme on HIV/AIDS (UNAIDS) and partners had the aspiration that 73% of people living with HIV would have viral suppression by 2030, a crucial step in ending the AIDS epidemic. This goal can be achieved by implementing the 90–90–90 targets, where the objectives are to diagnose 90% of all HIV-positive persons, provide ART to 90% of those diagnosed, and achieve viral suppression in 90% of those treated by 2020 [21]. Unfortunately, we had a 15.8% of follow-up loss, which reflects a critical deleterious variable that will impact our capability to reach the last goal of the HIV "90–90-90" care cascade. Therefore, strategies and programs to promote both treatment and follow-up adherence will be critical not only for HIV prevention, a crucial goal to end the AIDS epidemic but to stop kidney injury as well.

## Conclusion

Our study showed a higher prevalence of CKD (15.8%). Coinfections play an essential role in the development of CKD. Therefore, the identification and prompt management of CKD and coinfections, such as TB and HCV, should be considered to avoid the progression and to delay renal replacement therapy as long as possible.

A low CD4/CD8 ratio accurately describes an immune dysfunction and has been used as a biomarker for disease progression of several non-AIDS-associated pathologies, such as kidney disease, heart disease, neurocognitive disorders. Unfortunately, more than half of our patients were late presenters (with < 200 cells/μL), and only 7.8% had an optimal CD4/CD8 ratio. Hence, it is important to make earlier HIV diagnoses and provide timely treatment to avoid the progression of AIDS or non-AIDS diseases, including CKD and the need for dialysis. Finally, it is important to consider from the beginning the expected nephrotoxic effect of some drugs alone or in combination, coinfections, or after the combination of some of them and, to make the correct follow-up in these patients.

## Data Availability

All data generated or analyzed during this study are included in this published article.

## Abbreviations

HIV: Human Immunodeficiency Virus
CKD: Chronic Kidney Disease
OI: Opportunistic Infections
RR: Relative Risk
HBV: Hepatitis B Virus
HCV: Hepatitis C Virus
TB: Tuberculosis
ART: Antiretroviral Therapy
eGFR: Estimated Glomerular Filtration Rate
ACR: Albumin-creatinine Ratio
HIVAN: HIV-associated Nephropathy
CKD-EPI: The Chronic Kidney Disease Epidemiology Collaboration
ICD-10: World Health Organization's International Classification of Diseases
sCr: Serum Creatinine
BMI: Body Mass Index
KDIGO: Kidney Disease Improving Global Outcomes
IQR: Interquartile Ranges
SD: Standard Deviations
SPSS: Statistical Package for the Social Scientist
AIDS: Acquired Immunodeficiency Syndrome
CDC: Centers for Disease Control and Prevention
STR: Single-tablet Regimen
NNRTIs: Nonnucleoside Reverse Transcriptase Inhibitors
TDF: Disoproxil Fumarate
FTC: Emtricitabine
EFV: Efavirenz
INsTI: Integrase Inhibitors
ABC: Abacavir
3TC: Lamivudine
DTG: Dolutegravir
